# Editorial for the Special Issue “Antiprotozoal Activity of Natural Products”

**DOI:** 10.3390/antibiotics12121650

**Published:** 2023-11-23

**Authors:** Cecilia Baldassarri, Eleonora Spinozzi, Marta Ferrati, Paolo Rossi, Filippo Maggi, Riccardo Petrelli

**Affiliations:** 1 Chemistry Interdisciplinary Project (ChIP) Research Center, School of Pharmacy, University of Camerino, Via Madonna delle Carceri, 62032 Camerino, Italy; cecilia.baldassarri@unicam.it (C.B.); eleonora.spinozzi@unicam.it (E.S.); marta.ferrati@unicam.it (M.F.); 2 School of Biosciences & Veterinary Medicine, University of Camerino, Via Gentile III da Varano, 62032 Camerino, Italy; paolo.rossi@unicam.it

Neglected tropical diseases (NTDs), a diverse group of infectious diseases, represent the leading cause of morbidity and mortality among the world’s low-income populations. These diseases are often referred to as “neglected” due to their historical lack of attention from the global health agenda and the lack of research investments in this area, with the exception of important support from the Bill & Melinda Gates Foundation. According to the World Health Organization (WHO), over 1.5 billion people are affected by at least one NTD, causing around 500,000 annual deaths, social discrimination, and physical suffering. Although NTDs can be found worldwide, they are most prevalent in tropical areas, where factors such as limited access to clean water, favorable environmental conditions, and inadequate sanitation expedite their transmission. Among a core group of 20 NTDs and conditions, the infections caused by single-celled protozoan parasites, such as Human African Trypanosomiasis (sleeping sickness or HAT), Chagas disease, Leishmaniasis, and Malaria, are regarded as therapeutically challenging because of their elevated fatality rates and limited treatment options [1,2]. Even though Malaria is not included on the list of NTDs, it remains a significant public health issue in many NTD-endemic regions, and it is frequently considered within the framework of initiatives aimed at managing and eradicating NTDs [3].

Kinetoplastid infections are transmitted by insect vectors and kill approximately 15,000 people annually, making them one of the more lethal NTDs. Protozoal diseases mainly impact populations in economically disadvantaged countries and offer limited commercial attractiveness for pharmaceutical companies in terms of obtaining a return on their investments. However, significant progress has been achieved in the last 10 years in exploring alternative approaches to controlling and eliminating kinetoplastid infections, including identifying novel druggable targets and a robust pipeline of drug candidates, as well as several attempts to develop vaccines. Nevertheless, the current therapeutic options are based on expensive and often toxic medicines that are limited in quantity and require parenteral administration within resource-limited healthcare settings that are often characterized by the lack of qualified personnel. A potential solution could be the development of a preventive vaccine. However, overcoming the complexities associated with protozoan infections requires tailored approaches for each pathogen. Many protozoa have complex life cycles or exhibit antigenic variation by changing their surface proteins to evade the host immune response. This behavior makes it difficult to develop a vaccine prototype that provides long-lasting immunity. Overall, the situation emphasizes the critical nature of this phenomenon and the pressing need to investigate new sources of potentially effective and safe compounds for therapy. In this context, naturally occurring products may play a pivotal role as sources for bioactive drug candidates, given that they could offer several advantages over conventional synthetic-based drugs. (e.g., biocompatibility, better bioavailability, polypharmacology, less long-term toxicity, and evolutionary optimization).

On this basis, this Special Issue has been designed to gather a collection of original articles and review papers dealing with the potential antiprotozoal activities of plant secondary metabolites, their hypothetical mechanism of action, and the development of new antiprotozoal formulations.

The first section focuses on the potential of new naturally based antiprotozoal agents. Coy-Barrera and coworkers investigated a series of kaurane-type diterpenoids as potential inhibitors of the bifunctional enzyme Dihydrofolate Reductase-Thymidylate Synthase (DHFR-TS) [4]. DHFR-TS is a crucial enzyme for the parasite’s survival, making it an attractive target for anti-Leishmania chemotherapies. DHFR catalyzes the reduction of dihydrofolates into tetrahydrofolates, which is essential for maintaining intracellular folate levels. In trypanosomatids, DHFR and TS domains are fused into a single enzyme, making this process vital for purine and pyrimidine nucleotide biosynthesis. Instead, humans have separate DHFR and TS polypeptides, making DHFR-TS an attractive antiprotozoal target. Nevertheless, DHFR inhibitors were largely ineffective in controlling trypanosomatid infections, since the parasites acquire folate from hosts through pteridine reductase 1 (PTR1). This phenomenon might better explain why antifolate drugs have been ineffective in treating trypanosomatid parasites. In this study, the authors focused on four kauranes and two derivatives. Compounds **302** and **302a** emerged as potential inhibitors of both PTR1 and *Leishmania major* (*Lm*) DHFR-TS, with IC_50_ values of 6.3 and 4.5 µM, respectively. In order to evaluate the hypothetic mechanism of action of compounds **302** and **302a**, the authors conducted molecular docking calculations and molecular dynamics simulations using a hybrid model of DHFR-TS. The in silico studies revealed the crucial role played by the hydrogen bonds and the *p*-hydroxyl group of the phenylpropanoid moiety in inhibiting DHFR-TS. Furthermore, computational studies on *Leishmania* species that cause cutaneous and mucocutaneous leishmaniasis demonstrated that compounds **302** and **302a** are potential multispecies inhibitors. These findings provide a promising avenue for developing dual PTR1/DHFR-TS inhibitors as alternative chemotherapy strategies against *Leishmania* infections.

A study by Setzer and coworkers reported the chemical characterization and in vitro and in silico antileishmanial evaluation of the essential oil (EO) from *Croton linearis* Jacq. stems (CLS-EO) [5]. *Croton linearis* is an aromatic shrub with a history of traditional medicinal use in the Bahamas, Jamaica, and Cuba, known for its antiprotozoal properties. In this study, the authors aimed to identify the major components in CLS-EO and assess its in vitro antileishmanial activity. Gas chromatography–mass spectrometry analysis revealed a complex mixture of monoterpene hydrocarbons and oxygenated monoterpenes with 1,8-cineole (27.8%), α-pinene (11.1%), *cis*-sabinene (8.1%), *p*-cymene (5.7%), α-terpineol (4.4%), *epi*-γ-eudesmol (4.2%), linalool (3.9%), and terpinen-4-ol (2.6%) as the major components. CLS-EO demonstrated antileishmanial activity against both promastigote (IC_50_ = 21.4 ± 0.1 μg/mL) and amastigote (IC_50_ = 18.9 ± 0.3 μg/mL) forms of Leishmania, with a selectivity index (SI) of 2 and 3, respectively (peritoneal macrophages from BALB/c mice, CC_50_ = 49.0 ± 5.0 μg/mL). Motivated by the observed antiprotozoal potential of CLS-EO, the authors conducted a molecular docking analysis to shed light on the antileishmanial activity of the major EO components. *epi*-γ-Eudesmol emerged as a key metabolite, and the observed biological activity may arise from synergistic interactions among EO components. Finally, the authors suggested that multiple mechanisms of action could underline the observed antiprotozoal activity, and they encouraged further exploration of CLS-EO’s major components as potential antileishmanial agents.

Fatmi and coworkers investigated the antiprotozoal activity of thymoquinone (TQ) for the treatment of *Lm* [6]. TQ, a monoterpene found in *Nigella sativa* L. EO, demonstrated significant in vitro antileishmanial activity against both promastigotes (EC_50_ = 2.62 μM) and amastigotes (EC_50_ = 17.52 μM) of *Lm*, with a promising SI (11.27 and 1.69, respectively). Molecular docking highlighted squalene monooxygenase as the preferred target for TQ. Molecular dynamics simulations confirmed the stability of TQ in the binding pocket. Their study concluded that TQ is a promising naturally occurring alternative for tackling drug-resistant leishmaniasis.

Antimicrobial resistance (AMR) is becoming a growing global threat in both bacteria and pathogenic protozoa. To address this challenge, Szumny et al. [7] investigated a novel formulation comprising EOs (e.g., those from eucalyptus, lavender, cedar, and tea tree), organic acids (acetic acid, propionic acid, and lactic acid), and metal ions (Cu, Zn, and Mn). Protozoans, including *Pentatrichomonas hominis*, *Gregarina blattarum*, *Amoeba proteus*, *Paramecium caudatum*, and *Euglena gracilis*, were chosen as models for this study. The authors reported unexpectedly potent antiprotozoal activity in these combinations, and the efficacy of these novel mixtures exceeded that of conventional antibiotics, chloramphenicol, and metronidazole. Most of the formulations exhibited high antiprotozoal activity at very low concentrations (0.001 to 0.009%). Cedar and tea tree EOs, in combination with a mixture of acids and manganese or zinc ions, exhibited remarkable antiprotozoal properties at low concentrations (0.001 to 0.008%). To the best of our knowledge, this study is the first reported work to explore the antiprotozoal activity of combinations consisting of EOs, organic acids, and metal ions.

Motivated by the need for new, less toxic, cost-effective, and easily administrable treatments for HAT, Baldassarri and colleagues [8] delved into the examination of *Trypanosoma brucei* (*Tb*)’s susceptibility to *Anthriscus nemorosa* M. Bieb. EOs, isolated compounds, and artificial mixtures, both in conventional and nanoencapsulated forms. The authors first analyzed the chemical composition of *A. nemorosa* EOs from different plant parts. The *A. nemorosa* EO from the aerial parts exhibited significant antitrypanosomal activity, with an EC_50_ value of 1.17 μg/mL, and the root EO had an EC_50_ of 2.36 μg/mL. The study revealed that the major activity against *Tb* came from less abundant components, such as farnesene and *β*-ocimene, which showed equal or greater antitrypanosomal effects compared with the *A. nemorosa* EO. Based on these findings, the synergistic interactions among the EO’s components were investigated by building artificial mixtures (Mix 1-11) that encompassed all the major constituents. These mixtures were engineered to replicate the natural EO’s composition, excluding components that did not exhibit antitrypanosomal activity. The artificial blends (Mix 3–5) exhibited noteworthy potential as sources of antiprotozoal agents, with EC_50_ values spanning from 1.27 to 1.58 μg/mL. To overcome the limitations of EOs, such as instability and poor physicochemical properties, the researchers used nanoemulsions to enhance the stability and bioavailability of the EO from the aerial parts. While the nanoemulsions showed slightly higher EC_50_ values compared with the natural EO, they offered an improved SI against trypanosomes, making them a potential delivery system in regions with inadequate sanitation conditions. Finally, a potential mechanism of action of *A. nemorosa* EO from the aerial parts and roots has been hypothesized. The EOs disrupt the energy metabolism of *Tb*, leading to a decrease in nucleoside triphosphates (NTPs) and an increase in adenosine diphosphate (ADP) levels. This disruption indicates the impairment of adenosine triphosphate (ATP) production, which is vital for maintaining intracellular ion balance.

Schmidt and collaborators [9] reported on the isolation of 25 alkaloids from *Buxus sempervirens* L., including eight novel natural products and one previously unreported compound. A comprehensive structural elucidation was achieved using advanced analytical techniques. The isolated Buxus-alkaloids were screened against *Plasmodium falciparum* (*Pf*) and *Trypanosoma brucei rhodesiense* (*Tbr*). Additionally, their cytotoxicity against the L6 cell line (mammalian cells) was evaluated as a counter-screen for cytotoxicity. This study was inspired by a previous report on an isolated compound, O-tigloylcyclovirobuxeine-B from a *B. sempervirens* leaf extract, which exhibited potent and selective in vitro activity against *Pf*. Several compounds displayed noteworthy in vitro activity against these two pathogens, with IC_50_ values below 1.0 µM and a high SI. Notably, cyclomicrophyllidine-B (7) and O-benzoyl-cycloprotobuxoline-D (8) were the most active against *Pf*, with IC_50_ values of 0.2 and 0.18 µM, respectively. Furthermore, these compounds showed potent antitrypanosomal activities against *Tbr*, with the new natural product 8 again being the most effective compound (IC_50_ 1.1 µM and SI = 12). This research offers insights into the structural features that contribute to antiprotozoal activity and reduce the toxicity of the most active compounds, creating the framework for future structure–activity relationship studies. Finally, this work demonstrated that Buxus-alkaloids hold the potential to be used as novel antiprotozoal lead compounds.

The second section of this Special Issue includes an extensive review of next-generation human liver models for antimalarial drug assays by Kulkeaw [10]. In this review, the author discusses the ongoing global threat posed by Malaria, despite significant progress in prevention and treatment. Existing drugs targeting liver-stage parasites have limitations, particularly in individuals with a glucose-6-phosphate dehydrogenase deficiency. However, one major obstacle to developing new drugs has been the lack of suitable in vitro culture systems for evaluating drug efficacy and toxicity. Current culture systems primarily rely on immortalized or cancerous cells, which do not accurately represent the human body’s complex cellular architecture. Although primary human cells are more physiologically relevant, they face issues related to variability, limited supply, and ethical concerns. The review highlights the potential of pluripotent and adult stem cells for modeling liver-stage Malaria. Organoid models currently represent a major technological breakthrough, even though they have some limitations, e.g., the absence of essential stromal components such as immune cells and drug penetration issues due to their rigid extracellular matrix. Advances in stem cell technologies and multidimensional culture show promise in overcoming these limitations, offering a potential alternative for the preclinical phase of drug discovery. Furthermore, the review touches on the challenges of assessing drug efficacy and the need for alternative methods to measure liver-stage schizonts. The author suggests that high-content imaging and fluorescence signals from transgenic *Plasmodium* parasites may offer more efficient and less subjective alternatives.

In conclusion, this Special Issue collected six articles and a review that underlined the importance of exploring new natural products with antiprotozoal activity that hold great promise for the future of tropical medicine and global health. In the last two decades, significant progress has been made in identifying and characterizing natural products with antiprotozoal potential. A considerable number of these natural products have already found applications in drug discovery or served as lead compounds for further synthetic optimizations. There are several meaningful prospective opportunities for forthcoming research and development in the field of antiprotozoal natural products. For example, combining natural-based antiprotozoal agents with existing drugs may enhance treatment efficacy while mitigating the risk of drug resistance. Alternatively, elucidating the precise mechanisms of action of promising natural scaffolds could offer valuable insights into the vulnerabilities of the protozoan machinery and help develop targeted therapies. Lastly, developing plant-based vaccines represents a promising strategy for fighting protozoal diseases. Plant-based vaccines involve using plants as bioreactors to produce antigens or proteins that can stimulate an immune response in humans. These antigens mimic the infectious agents that cause protozoal diseases, and when administered, they prompt the immune system to develop immunity against the pathogens. Currently, the development of plant-based vaccines for protozoal diseases is constantly evolving and is making progress, but several challenges remain. These challenges include optimizing production methods, ensuring vaccine safety and efficacy, and successfully overcoming regulatory hurdles for approval and distribution. Although challenges remain, the commitment to tackling protozoal diseases through vaccination initiatives is steadily increasing.
